# Supplementary material for: Echocardiography Nomogram for Predicting Survival among Chronic Lung Disease Patients with Severe Pulmonary Hypertension
Source: J Clin Med. 2022 Mar 14;11(6):1603. doi: 10.3390/jcm11061603 (PMC8955171; doi:10.3390/jcm11061603)
Supplement: Supplementary file 1 [file jcm-11-01603-s001.zip › jcm-1568101-supplementary.pdf]

Table S1. Comparisons of pulmonary function test in the derivation cohort.

	Obstructive lung diseases<br>*n* = 55	Restrictive lung diseases<br>*n* = 5	Other diseases with mixed restrictive/and obstructive pattern<br>*n* = 4	Hypoxia without lung diseases<br>*n* = 3
FEV1% pred	30.9 (22.7, 44.4)	71.7 (50.6, 103.9)	47.7 (26.5, 79.3)	46.3 ± 36.9
FVC % pred	54.3 (40.9, 80.9)	95 (60.6, 109.2)	61.0 (45.7, 90.6)	70.8 ± 25.3
FEV1/FVC %	48.8 (42.3, 57.0)	81.7 (50.4, 86.6)	63.1 (46.4, 73.4)	49.2 ± 22.6
RV % pred	210.4 (151.6, 270.8)	119.6 (106.8, 180.4)	134.4 (107.8, 158.7)	208.1 ± 103.9
TLC % pred	115.9 (95.0, 139.0)	109.4 (105.5, 115.0)	88.5 (84.3, 95.4)	122.5 ± 26.0
DLco % pred	36.4 (25.4, 54.1)	54.2 (21.1, 75.6)	36.8 (29.3, 50.9)	36.2 ± 6.0

Pred: predicted; FEV1: forced expiratory volume in 1 s; FVC: forced vital capacity; RV: residual volume; TLC: total lung capacity; DLco: diffusing capacity for carbon monoxide.
